# Notes of an European Tour

**Published:** 1845-10-01

**Authors:** F. H. Hamilton

**Affiliations:** Professor of Surgery in Geneva Medical College


### BUFFALO MEDICAL JOURNAL

**Vol. 1.** OCTOBER 1, 1845. **No. 5.**

### Notes of an European Tour.---No. 5.

Communicated for the Buffalo Medical Journal, by F. H. HAMILTON, M. D., Professor of Surgery in Geneva Medical College.

DEAR M. SWITZERLAND.

During my short stay at Geneva I went often to the hospital, and I am certain nothing can exceed the system and cleanliness of Dr. Lombard's fever wards. The salles are well ventilated; each patient has two beds to enable him to change occasionally from one to the other, the linen is renewed daily and kept perfectly smooth lest folds should produce bed sores, large pieces of buckskin being also sometimes placed underneath the sacrum, the teeth and mouth are carefully moistened and kept clean. by brushes made for the purpose, and with the view of still farther promoting cleanliness, an object of such importance in fevers, the head is in nearly all grave cases, entirely shaven. Among the therapeutic means to which Dr. L. resorts in *typhoid* fever, I may enumerate especially, frequent tepid or cold baths, venesection, tonics, and calomel in alterative doses, which latter, until lately, has not been much employed in Switzerland. Dr. Lombard has devoted much attention to the investigation of typhoid and bilious fevers, both of which prevail considerably during certain seasons at Geneva and in its vicinity, and his various publications upon these subjects, together with several other highly valuable reports and essays which he has kindly presented to me, I should be glad to see translated and published at home.

With regard to Geneva as a residence for invalids, I will make a simple statement of facts, since upon this point some difference of opinion seems to exist, and because Geneva has frequently been selected as the most suitable place on the Continent for the education of American Protestant Youth. At Geneva tables of death have been regularly kept since 1660! and M. le docteur d'Espine in his report for the year 1842 declares the mortality for that year in the Canton, including a population of 60,000, of whom about one half belong to the city, to be one in forty-seven and a half, which is precisely the mortality of your own city [Rochester, N. Y.] during the same year, and nearly the same with Boston. The average of deaths from *pulmonary* affections [upon which point the dispute has chiefly arisen] during the year 1842 was 25.8 per cent., while the average in your city in the same year was 30.85 per cent., and in Boston nearly 33 per cent. I have chosen the year 1842 simply because I possessed the means of instituting a comparison between these three towns on this year. The reports for five years in the Canton show about the same average. The rate of life here presented I should also add is nearly double that of Amsterdam in Holland [1 in 24] and of Rome in Italy, [1 in 25] while at Brussels it is 1 in 26, at Naples 1 in 28, Paris and Lyons 1 in 32, Leghorn 1 in 35, Palermo and Nice 1 in 37, and even at Glasgow, so much celebrated for its high range of life, 1 in 44. In short it is higher than in any European town of its size with which I am acquainted.

The morning I left Geneva I went again to the Cemetery "Plein Palais" to see the tomb of Sir Humphrey Davy, who died suddenly in 1829 at the "Hotel de la Couronne" and is buried here, but the gates were not open and I could not wait. On my way back I made a slight detour to get a look at the ramparts and the old prison house in which the honest but simple hearted Dr. Bobzee composed and sang verses while suffering imprisonment for having taught the heresy of universal salvation, and from which he would certainly have been led to the scaffold had it not been for the interference of the Protestant Churches in other Cantons. "Oh *Christianity*, what crimes have been committed in thy name!" Between 1592 and 1652 one hundred and fifty-four citizens of Geneva were executed and their property confiscated for the crime of witchcraft, denominated "*lese majeste divine au plus haut chef.*" During the prevalence of the plague in 1615, when about one third of the population had died in six months, a cry having arose that certain wicked persons were spreading the contagion, thirty-one were seized and upon confessions extorted by the torture were burnt at the stake, among whom was one Surgeon whose execution was signalized by the most barbarous cruelties. He was torn with pincers and while yet alive drawn into quarters by four horses, "*tenailles et ecartelles!*" Several heads of families who fled from the pestilence, were fined to the amount of 3872 florins, for the benefit of those who remained. Franklin used to say when contagious diseases prevailed, "go soon enough, go far enough, and stay long enough," but the Geneva sages thought differently.

At eight o'clock I went on board the "Aigle," one of the three or four very comfortable little steamers which now ply between Geneva and the towns above. The day was charming, and the lake, unmoved by a ripple reflected from its polished surface the villas, hamlets, spires, trees and rocks which line its shores, and even the more distant mountains were distinctly mirrored upon this beautiful camera; a picturesque looking craft with steep prows, tall, slender masts and lateen sails, lay sleeping here and there upon its bosom, and it was pleasant from over the taffrail to catch the merry laugh and wild guttural song of the fishermen and fisherwomen, as together, and with measured strokes, they pushed the light "chaloupe." We had also upon deck our own band of wandering Swiss minstrels, with their guitar and violin, and alpine voices, and noble faces, and graceful native costumes, and the small tin box to rattle a "batzen" in, when the music was done.

At Coppet we stopped a few minutes to exchange passengers; to the rear of the town stands a chateau now occupied by the "Duc de Broglie," formerly the residence of Necker and his daughter, Madame de Stael, both of whom are buried in the garden attached to the house. Stopping successively at Nyon, Rolle and Morges, we came next to Ouchy. The ancient Lousonne stood near where Ouchy stands now, but it was overwhelmed and destroyed 1300 years ago by a sudden rise of the lake.—The present town of Lausanne, as it is now spelt, and Capitol of the Canton Vaud, is built about a mile back from the shore, upon the side of a lofty and broken hill. It has a population of 1400, and boasts among its "sights" the house in which Gibbon, the "lord of irony," wrote his "Decline and Fall of the Roman Empire;" and also another, the residence of Rosseau. I should have been equally interested to have visited the excellent hospital under the care, I believe, of M. Mayor.

Lausanne is frequently selected by invalids and families of wealth and pleasure as a summer residence. Those subject to pulmonary diseases are particularly warned however, not to expose themselves above the town during the prevalence of the "vent de bise," a peculiarly sharp north east wind.

William Fabricius, surnamed Hildanus, an eminent surgeon and anatomist, was in 1582 a resident of Lausanne: and Haller publised here in 1757, after thirty-six years of unremitted labor, his remarkable work entitled "Elementia Physiologia Corporis Humani" in eight quarto volumes. It is to-day nearly a century since here upon the mountains of Lausanne, Haller laid the corner stone of the doctrine of Physiological Pathology, to which Bichat and his successors have added the superstructure, and which at the return of its first centennial cycle holds entombed beneath its massive fabric, all those mechanical, chemical, humoral and physiological phantasms which for so many centuries preceding had occupied the minds of medical men.

Vevay is delightfully situated toward the upper part of the lake, the shores just beyond rising rapidly to an unusual height and being clad to their very summit with terraced vineyards. At some places I counted from 50 to 60 of these terraces rising one above the other, and built with incredible labor along the precipitous sides of the mountain. Above the town is seen the fine chateau and grounds of M. D'Hauteville, whose marriage and subsequent divorce from our fair countrywoman, a few years since occasioned so much remark and gossip. He is now in his second honey-moon, having lately married a young and beautiful German Lady of large fortune. My informant, a Gentleman from Geneva going to Vevay, declared that to every one here, the conduct of his former wife was a matter of surprise, since to all the accomplishments of a Gentleman, "amiable, excellent, and intelligent," M. D'Hauteville added the attractions of a princely estate in the highest state of cultivation, his gardens being the frequent resort of travellers, and his chateau commanding one of the finest views in the world. You remember Rosseau's apostrophe to the scenery around this place, in which he thus concludes: "I must absolutely have an orchard near the border of this lake and of no other. I must have a true friend, an amiable woman, a cow and a little boat. Never upon the earth shall I enjoy perfect happiness, unless in the possession of all these." But it was the "amiable woman" which, both M. D'Hauteville and Rosseau, found it most difficult to obtain.

In the Church of St. Martin at Vevay, is buried Ludlow, who executed the parliamentary sentence upon Charles the first. Upon the restoration of Charles the second a price was set upon his head, and he fled to Vevay for refuge, where he remained until his death. Charles the first was a wonderful Doctor, as well as a most profligate King in his day, having cured no less than 23,000 persons of the Scrofula by his "tactus regalis," not to speak of the well attested cures effected by his *blood*, scraped up upon chips after his "inhumane decollation by the barbarous regicides." We ought never to forgive Ludlow for depriving our profession of so kingly an ornament. Vevay has a hospital, founded in 1794.

About 3 o'clock P. M. we were landed at Villeneuve, situated near the head of the lake, in the midst of some of the wildest Swiss scenery. Having taken a brief lunch at l'Hotel de Ville, and learning that I could not leave for the "Valais" until four the next morning, I determined to walk to Chillon, only one mile distant. Leaving Villeneuve from the west, I followed the road which winds along the northern shore of the lake towards Vevay and Lausanne. Scarcely a mile from Villeneuve I crossed a small but noisy mountain torrent, fed by the glaciers of Arvel and La Tiniere whose summits, seen on the right many thousand feet above, send tribute to the German Ocean on the north, and the Mediterranean sea on the south. A few rods beyond, the spreading base of the mountain projects into the lake forming an abrupt promontory, around and underneath which the road is hewn; directly opposite, and separated from the road only by a narrow strait, upon an insulated rock rising abruptly from the bottom of the lake, stands the lone Castle of Chillon; commanding thus most completely what was once the only entrance to and from the upper Rhone. This strong, and before the introdnction of fire arms, almost impregnable castle was built more than 600 years ago by one of the Dukes of Savoy, and from the peculiar strength of its walls and of its position, it was long used by the Savoy Kings as a prison house and tomb for all political and religious offenders. When in 1348 the "black plague" as it was called from its terrible fatality, was desolating the world, it was in this castle that the persecution of the unfortunate jews commenced; accused by a terrified and superstitious populace of having poisoned the wells, and thus being the authors of their present calamities. It was in an upper room, called the "hall of justice," that in the month of September 1348, the first accusations and mock trials occurred—and it was in the vaults below, that the torture was first applied, and the executions and living burials first occurred, which, to the eternal disgrace of humanity, were soon repeated in horrible detail and variety throughout Switzerland, and almost throughout Christendom. Chillon was also a prison for the early Reformers and Swiss Patriots, and when in 1536 the Bernese, driven to desperation by the cruelties and usurpations of Charles of Savoy, besieged and took this castle, the last hold of the Duke, Bonnivard, a noble Genevese patriot who had suddenly disappeared six years before while crossing the Jura mountain, and many other captives, were found immured within its dungeons.

Crossing the draw bridge which connects the Island with the main land, I saw standing within the gate of the outer wall a Swiss girl who was evidently watching my approach. She had flaxen hair and jolly blue eyes, with a complexion rich and ruddy as the alpine rose; her slight form was moreover most astistically displayed in the national peasant costume. I had not expected to see such a flower in the midst of these rocks, and much less at the gate of the old castle of the iron duke of Savoy. The bunch of massive keys however, which she sported from her belt, assured me that she was both porter and guide, and I

addressed her accordingly. "Yes sir" said she "I am the conciege to-day; my brother David is at work about the Castle and I will go with you. I was not sorry I must confess that to-day David had other work since such a companion would somewhat abate from the awe which these gloomy walls had already inspired. My pretty guide stepping lightly before me led me first through a second gateway into a small base court, and from thence, having provided ourselves with a lamp, we descended by 14 rude stone steps, to the lower range of prisons, all of which roughly hewn from the solid rock are scarcely above the level of the lake. Passing through the two first apartments, the "vestibule" and the "salle du garde," the first 10 by 20 feet in diameter, and the second 20 by 50, each supported by stone columns, and without light except what was admitted at the doors; we came then to the "salle du torture," a room 15 by 20 feet in diameter, into which a straggling ray of light entered through a small recessed window, or rather "arrow slit" in the water wall. Close against the opposite wall, hewn from the rock at an an- angle of 45 degrees with the horizon, was an inclined plane, wide enough and long enough to extend a man at full length; this was the "stone of torture:" and at the foot was a hollow, perforated below, and "used," said my guide "to receive and carry off the blood." The next room was smaller and wholly without light; on the north side I felt my way into a dark, damp and deep recess, in which the rocks were too irregular and slippery to enable me to stand securely without embracing the wall. Holding up the lamp, a heavy black timber could be seen traversing the top of the vault, and this was the "gallows beam." Opposite, in a niche, formerly stood (now removed) an image of the Virgin Mary lighted by a wax taper, to which the last prayers and confessions of the dying prisoner were offered. Immediately underneath this niche is a large square hole, closed by a stone door, through which the bodies loaded with heavy weights were thrown into the lake, which against this wall is several hundred feet deep. The opening into the next room was not higher than my shoulders and very narrow, secured by an oaken and iron bolted door, with a strong and curiously constructed lock. Pushing the door aside with both hands, for my petit guide was unable to move its rusty hinges, we were in the "grand dungeon" or principal room of confinement. This apartment is 76 by 20 feet; five small "arrow slits" in the wall toward the lake, are too high to allow the craggy summits of "Craux du Noe" or even the snowy aiguilles of "Dent d'Oche" to be seen from within, the latter of which rises directly opposite Chillon, to the height of more than 7000 feet. It is snpported through the centre by eight gothic stone columns, upon one of which I read, engraved with a chisel, the names of Abercrombie, Harvey, Peel, Byron &c., and upon another the name of Victor Hugo with many others. Near the base of a column at the west end, hangs fastened by a staple, a rusty iron ring, to which Bonnivard is said to have been attached; where it falls against the column it has worn a deep indentation; "this iron is a cankering thing." A smooth line extending towards thenearest light and sunk two or three inches in the solid floor was worn, "as if the cold pavement were a sod," by the dragging of his chain during the six long years of his confinement. The two adjoining columns on either side, have similar fastenings, to which Byron in his affecting poem entitled "Prisoner of Chillon" has supposed the two brothers of Bonnivard to have been chained.

From the vault I was led up to the second story, now occupied chiefly as an arsenal for the republic of Vaud, a portion of which is however, undergoing repairs to be again converted into a State Prison. In the third story is the "hall of justice" and the private apartments; when you visit Chillon you will find my name, not shut up in those cheerless dungeons below, but emblazoned directly over the comfortable looking, old fashioned fire place in the "sleeping room" of the Duke; the Swiss girl with saxon hair, has promised it shall not be disturbed. My name and "home" duly registered, I was shown into the chapel, and passing thence through a long gallery, we came to two or three small, well secured dungeons, constructed in the very thickness of the northern wall, one of which, called the "oubliette" or dungeon of death, has in the middle of the floor a small trap. My guide directed me to look down, but I could see only a dark hole like a deep and narrow well; then dropping a stone she requested me to listen, and I could hear it strike at first plainly against the rocks, but soon it became less and less distinct, until at length the sound ceased entirely, as if lost in the distance. It is said to communicate with the lake, and to have been one of the places where prisoners were secretly dispatched. But enough of these horrid mementoes of feudal ages! I have given you all in detail because it is the first of these castellated dens which I have carefully explored, and it is probably the last I shall attempt particularly to describe. The "oubliette" and the usual douceur of a couple of francs, satisfied both me and my guide, and we parted at the outer gate.

Crossing the moat I turned again to the left, and glad to breathe once more the air of freedom, walked hastily away towards Clarens, the village immortalized by Rosseau. The sun was down when I entered Montreux, a small hamlet situated partly in a gorge at the foot of "Dent de Jaman" and partly along the sides of the mountain. This place is remarkable for its salubrity. According to the statistics of Sir F. d'Invernois, *no place in the world* has so small a proportion of deaths, nor of "imprudent marriages!" I shall not fail to recommend it therefore both to invalids and to parents having marriageable daughters. Upon an eminence a little back of the town, occupied by a protestant congregation, stands the village church, with its tall New England spire. The bell was just ringing for an evening service, and I stopped a moment to listen to its soft, silvery notes as they fell from among the hills and were echoed from across the still lake.

A mile farther brought me to Clarens, where, with an old man whom I accidentally met and employed as a guide, I wandered about until a late hour, visiting such spots as "La Nouvelle Heloise" had memorialized and consecrated, and was about to turn towards Villeneuve when we encountered a gentleman to whom my guide introduced me as a "Countryman." I could not decline his pressing invitation to stop at his house a few moments and take with him a glass of wine, and I soon learned that my "countryman" was a young *Englishman*, without family, and who seemed living here in this out of the world place, as a mere matter of amusement; his name is Tanner. Learning that I was an American and a physician, he sent a messenger for his friend Dr. Boinzod, the village physician, who is a fine looking and exceedingly well informed young man. With cigars etc. we spent an hour together as if old and boon companions had met.—The Doctor, a graduate of the Parisian school, had been in practice but a few years, yet he had already made some clever operations, among which a shoulder amputation and an extirpation of the superior maxilla, Mr. Tanner himself had witnessed. Pthisis, he informed me, was rare in this neighborhood, but goitre and scrofula common. He had never extirpated a goitrous tumor, considering iodine, good air, and wholesome food capable of accomplishing all that belonged to our art. I was curious to know how he managed to attend to the peasantry among the mountains. "My beast has a very sure foot" said he, "and will carry me all day without a morsel to eat, and if a snow storm comes on he finds his way home by instinct. It is seldom that I am obliged to dismount, for he can generally climb where I cannot with safety. Sometimes indeed, it is impossible to reach the huts upon the mountains, but the inhabitants of such places are generally so hardy that they seldom suffer in consequence. As to pay, it is seldom money, occasionally a few bushels of chesnuts or some wild game, perhaps a chamois."

At 11 o'clock I started for Villeneuve, four miles distant, both of the gentlemen accompanying me about half a mile, and they would have gone farther but that I insisted upon their return; a mile or two beyond which the road, shut in for some distance by a high wall toward the lake, and overhung with rocks and thick forest trees upon the left, looked the very place for midnight robbers and assassinations. The moon shone brightly, but it only served to cast the stronger shadows across the road and along the deep ravines which every now and then sank away among the mountains; and I confess that apprehensions heightened perhaps by the report of a robbery recently committed by a bandit in the adjoining Canton, rendered my nerves unusually active and my steps more light and nimble than ordinary, and that I felt a sensible relief when the gray turrets of Chillon appeared again in sight.

The door of my Hotel was open when I reached Villeneuve, but not a soul was stirring. Having stumbled my way up the stone steps into the coffee room I found a light with a good supply of bread, cold ham, schrabzieger cheese and a bottle of Vevay wine, which mine host, anticipating my return, had not forgotten to set for me. The Vevay wine is a white and sweet wine and had to me a most excellent bouquet; the cheese and bread were superlative. I did not care to sleep, and indeed had I chosen, it would have been difficult to have found a place, for I had already made noise enough in getting up stairs and in stumbling over a wooden water pail, to awake the seven sleepers, but not the stirring of a mouse could be heard. Accordingly, after my last draught at the Vevay cordial, I drew out my note book and writing utensils and amused myself until daylight in "scoring up."

